# Surface wind mixing in the Regional Ocean Modeling System (ROMS)

**DOI:** 10.1186/s40562-017-0090-7

**Published:** 2017-11-02

**Authors:** Robin Robertson, Paul Hartlipp

**Affiliations:** School of Physical, Environmental, and Mathematical Sciences, University of New South Wales Canberra, Canberra, Australia

**Keywords:** Surface mixed layer depth, Wind-driven mixing, Vertical mixing parameterization

## Abstract

Mixing at the ocean surface is key for atmosphere–ocean interactions and the distribution of heat, energy, and gases in the upper ocean. Winds are the primary force for surface mixing. To properly simulate upper ocean dynamics and the flux of these quantities within the upper ocean, models must reproduce mixing in the upper ocean. To evaluate the performance of the Regional Ocean Modeling System (ROMS) in replicating the surface mixing, the results of four different vertical mixing parameterizations were compared against observations, using the surface mixed layer depth, the temperature fields, and observed diffusivities for comparisons. The vertical mixing parameterizations investigated were *Mellor*–*Yamada* 2.5 level turbulent closure (MY), *Large*–*McWilliams*–*Doney* Kpp (LMD), *Nakanishi*–*Niino* (NN), and the generic length scale (GLS) schemes. This was done for one temperate site in deep water in the Eastern Pacific and three shallow water sites in the Baltic Sea. The model reproduced the surface mixed layer depth reasonably well for all sites; however, the temperature fields were reproduced well for the deep site, but not for the shallow Baltic Sea sites. In the Baltic Sea, the models overmixed the water column after a few days. Vertical temperature diffusivities were higher than those observed and did not show the temporal fluctuations present in the observations. The best performance was by NN and MY; however, MY became unstable in two of the shallow simulations with high winds. The performance of GLS nearly as good as NN and MY. LMD had the poorest performance as it generated temperature diffusivities that were too high and induced too much mixing. Further observational comparisons are needed to evaluate the effects of different stratification and wind conditions and the limitations on the vertical mixing parameterizations.

## Background

The depth of the surface mixed layer plays a key role in ocean–atmosphere interactions, specifically the dissolved gases and the transfer of heat and fresh water balance from precipitation and evaporation, impacting the temperature and salinity fields in the upper ocean. The primary driving force for the surface mixing is the wind and the resulting waves and Langmuir circulation. The ability of ocean models to replicate the mixed layer and its depth is critical to providing the physical dynamics in support of chemical and biological simulations.

In models, the surface mixed layer depth is controlled by the vertical mixing parameterization. Presently, several vertical mixing parameterizatoins are being used. For example, the Regional Ocean Modeling System (ROMS) provides three of the most widely-used vertical mixing parameterizations, *Mellor*–*Yamada* 2.5 level closure scheme (MY) (1982), the *Large*–*McWilliams*–*Doney* Kpp scheme (LMD) (Large and Gent 1999), and the generic length scale (GLS) (Umlauf and Burchard 2003; Umlauf et al. 2003), which is often referred to as the general ocean turbulence model (GOTM). The GLS parametrization has four different flavors: *k* − *kl, k* − *ε*, *k* − *ω*, and generic, where *k* indicated the turbulent kinetic energy, *l* the dissipation scale, *ε* dissipation, and *ω* the inverse turbulent time scale.

The question is which one of these performs the best for wind-driven mixing. Although Durski et al. (2004) investigated the performance of MY and LMD in ROMS, a comparison was not made of the performance of the new GLS scheme. Burchard and Bolding (2001) investigated several flavors of GLS, but not specifically for ROMS. Others that have investigated the effects of the vertical mixing parameterizations under specific conditions, include Wijesekera and Gregg (2003) and Price et al. (1986). Price et al. evaluated the surface mixing and the diurnal heating cycle, which is of interest here; however, the vertical mixing parameterizations and models have made significant improvements since then. We were interested in the surface mixed layer during the diurnal heating/cooling cycle with winds. Here, we investigated the ability of ROMS to replicate the wind-generated mixed layer and the vertical temperature diffusivity against observations for four vertical mixing parameterizations: MY, LMD, GLS: generic, and *Nakanishi*–*Niino* (NN) (2009). With the exception of NN, these are presently the most widely used parameterizations for vertical mixing. NN is an improved version of MY, specifically designed to better simulate upper ocean mixing and was implemented in ROMS by the authors.

## Methods

Simulations were performed using the regional ocean model system (ROMS) using four different vertical mixing parameterizations at four observational sites: one in deep water with typical temperate stratification (FLX91) and three in shallow water with weak stratification in the Baltic Sea. This paper will concentrate on the FLX91 program carried out during May of 1991 in the Eastern Pacific off Northern California for brevity. The mixing schemes investigated were: *Mellor*–*Yamada* 2.5 level turbulence closure scheme (MY) (Mellor and Yamada 1982), the KPP scheme developed by *Large*–*McWilliams*–*Doney* (LMD) (Large and Gent 1999), the Generic Length Scale scheme (GLS) developed by Umlauf et al. (2003), and Nakanishi and Niino’s vertical mixing parameterization (NN) (2009). Observational sites were selected both due to the extensive data sets available there, including wind and solar radiation measured at the site, and the ability to isolate wind forcing. They were also in areas with fewer eddies and/or fronts passing through, particularly the FLX91 site in the Pacific Ocean. Eddies and fronts shift the mixed layer depth independently from the wind. These sites had high-resolution observational data and coincident winds. Both the FLX91 and Baltic Sea sites have extensive data sets that include observations of vertical mixing through microstructure profilers, which enabled a more complete evaluation.

The FLX91 experiment was conducted in the Eastern Pacific off Northern California by Jim Moum’s group at Oregon State University during 1–7 May 1991 (Hebert and Moum 1991; Moum 1996a, b). They used a free-falling Chameleon to collect pressure, potential temperature, conductivity, temperature gradient, horizontal velocity shears, and vertical velocity fluctuation data. Chameleon drops were repeated at roughly 10 or 20 min intervals over a 6 day time period. Wind, air temperature, humidity, solar radiation, ADCP velocity measurements came from the on-board instruments (Hebert and Moum 1994). Heat flux and surface stress were calculated from bulk formulas (Hebert and Moum 1994). CTD data were used for the depth below the reach of the Chameleon, ~ 300 m. However, since we were primarily interested in the surface mixed layer, the Chameleon profiles covered our area of interest. Estimates of dissipation,*ε*, and vertical diffusivity, *K*_*ρ*_, were determined from the Chameleon data. For our purposes, the Chameleon drops in one area were treated as if they were a time series of data at one point. The complete data set, *ε* and *K*_*ρ*_, was kindly provided by Jim Moum.

**Fig. 1:**
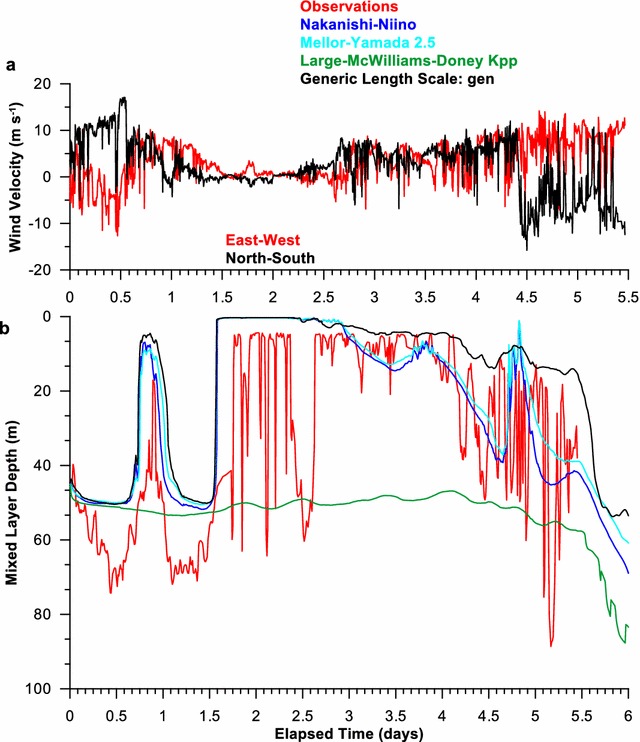
**a** The East–West (red) and North–South wind velocities during the FLX91 experiment. **b** The smld as determined for the observations (red) and the model results for the *Nakanishi*–*Niino* (blue), *Mellor*–*Yamada* 2.5 (cyan), *Large*–*McWilliams*–*Doney* (green), and GLS (black) vertical mixing parameterizations

Two storms passed over the site during Chameleon observations, days 1 and 4 (Fig. 1a). Both diurnal and semidiurnal tides were present in the observational data set and dominated the velocities in the upper water column (< 150 m) Hebert and Moum 1994); consequently, tidal forcing was used in these simulations. The tidal velocities were predominantly barotropic and did not add significant vertical shear to the horizontal velocities (Hebert and Moum 1994).

A set of three flux experiments was carried out in the Baltic Sea in ~ 55 m of water during early September 2001 (30 Aug-7 Sept), late June 2002 (26–30 June), and early October 2002 (2–9 October). The data from these experiments were kindly provided by Lakshmi Kantha and included potential temperature, salinity, vertical shears in the horizontal velocities, and dissipation at roughly hourly intervals at 0.5 m vertical resolution. These data were collected primarily using microstructure profilers, but CTD data were also collected in the region. Meteorological data, solar radiation, surface temperature, and surface salinity from on-board observations were provided at hourly intervals. The tides in the region were very weak, so tidal forcing was not included in these simulations. Salinity is very low in this region and the region is relatively devoid of eddies. More details on the data are given in the cruise reports (Prandke 2002a, b, c; Lass 2002a, b, c).

To evaluate the model performance in replication of the surface dynamics, ROMS simulations with the same wind stress were performed with the four different vertical mixing parameterizations: NN, MY, LMD, and GLS. Tides and solar radiation were included, except for tides in the Baltic Sea where they are negligible. The winds were ramped up during the first day. Background hydrography was taken from observations, as described for the sites, and was initially horizontally uniform over the domain. Simulations were performed at a nominal horizontal resolution of 1 km. The basic cases had 100 vertical levels for the Eastern Pacific in deep water and 50 levels for the Baltic Sea in shallow water. This resulted in a vertical model resolution in the upper ocean less than 2 m for the FLX91 and less than 1 m for the Baltic Sea cases. Additionally, to evaluate the effect of the vertical resolution, simulations were performed with 50 and 25 levels for FLX91 and 25 levels for the Baltic Sea. The results were saved at 10 min intervals over the 6 day simulations for evaluation.

There are some caveats with this approach. Several environmental considerations were not taken into account, including eddy dynamics, the passage of fronts, non-geostrophic currents, upwelling, far-field internal tides and waves, among other phenomena. Additionally, since the observational data sets extended for only a few days, the simulations were run for only 6 days.

## Results

To evaluate the performance of the four different vertical mixing parameterizations, model results were compared against time series of the observed data for potential temperature (*θ*), salinity (*S*), the East–West and North–South horizontal velocities (*u* and *v*, respectively), and temperature diffusivity (*K*_T_). To reduce the number of figures in this document, we will focus on potential temperature, *θ* and the temperature diffusivity, *K*_T_ or *K*_*ρ*_. Since the vertical temperature diffusivity depends on the stratification and vertical shears in the horizontal velocities, the *Brunt*–*Väisäla* frequency, *N*, the vertical shears, *U*_Z_ and *V*_Z_, the turbulent kinetic energy, *k*, and the length or dissipation scale, *l*, were also investigated. The key criteria for the evaluation were the depths of the surface mixed layer, the potential temperature field over time, and the vertical temperature diffusivity. The surface mixed layer depth, smld, was defined as a 0.2 °C difference from the surface, i.e. a reference depth of 0 m (Fig. 1b).

During the FLX91 experiment, the winds were strong the first day, weak during day 1.5–2.5, then building to 15 m/s (30 kts) during the second storm from day 2.5 to 5.5 (Fig. 1a). Since the winds were being ramped up during day 1 in the simulations, the strong winds of day 1 did not induce as much mixing as they would have at full strength and the day 1 response should be ignored.

The observed surface mixed layer depth (smld) (red in Fig. 1b) was a few meters deeper than that of the model results for NN (blue), MY (cyan), and GLS (black) and generally shallower than LMD (green). The general trend for the observed smld was matched by NN, MY, and GLS, but not LMD. The LMD smld was too deep and too constant to match the observations. The LMD smld was roughly around 50 m by 0.5 days and remained at that depth until 4 days. The smld for the other schemes were less than 20 m and so were the observed smld (Fig. 1b). Around 4 days, the wind strengthened suddenly, and the observed smld increased rapidly to fluctuate around 30 m. The fluctuations were strong, primarily ranging from the surface to 60 m, but once reaching 90 m. The smld’s of all mixing schemes increased at this time, but those of LMD dove ~ 20 m deeper than those of the other mixing schemes. LMD was also a bit slower to respond to the stronger winds. GLS was a bit too shallow and did not match as well as MY or NN. Neither MY or NN went as deep for the deep excursions as the observations. None of the vertical mixing parameterizations responded as quickly as the observations, although the simulation results were output at the same time interval as the observations.

**Fig. 2:**
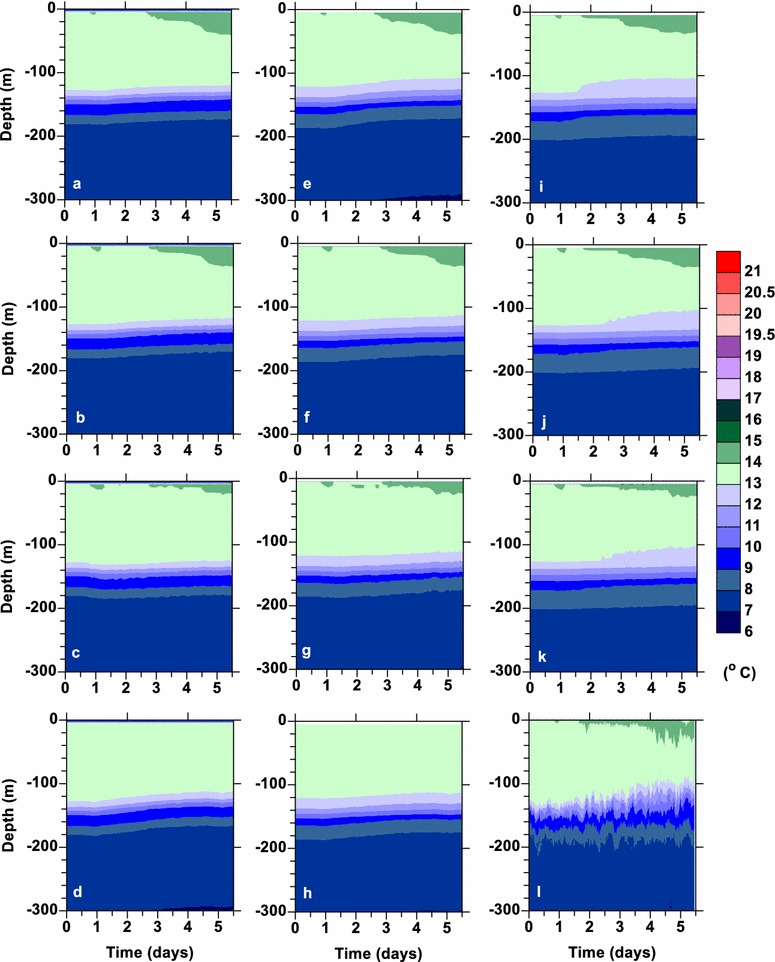
Potential temperature with depth during the FLX91 simulations (**a**–**k**) and the observations l. The simulations were done with (**a**–**d**) 100 levels, (**e**–**h**) 50 levels, and (**i**–**k**) 25 levels. The results from *Nakanishi*–*Niino* are given in (**a**, **e**, **i**), those from *Mellor*–*Yamada* 2.5 in (**b**, **f**, **j**), GLS in (**c**, **g**, **k**) and *Large*–*McWilliams*–*Doney* in (**d**, **h**). No results are shown for *Large*–*McWilliams*–*Doney* for 25 levels as they were essentially the same as those for 50 levels

Inspection of the potential temperatures from the model simulations (Fig. 2a–k) compared to the observations (Fig. 2l) shows that NN (Fig. 2a, e, i) and MY (Fig. 2b, f, j) simulated the observed potential temperature structure the best. LMD (Fig. 2d, h, 25 levels not shown) mixes the water column too much and does not show the warm surface layer forming like the other parameterizations. Note the results from the 25-level simulation for LMD are not shown as they were nearly identical to those with 50 levels. GLS (Fig. 2c, g, k) does not mix the warm surface water as much as occurred in the observations.

The vertical resolution did not appear to make much difference for the surface 40 m; however, the thermocline at 120–190 m thickened with fewer levels (Fig. 2). The potential temperatures with the coarse vertical structure, 25 levels, (Fig. 2i–k) actually matched in the thermocline region better than the higher resolution simulations, 100 and 50 levels, (Fig. 2a–h). The salinity structure between the simulations was essentially equivalent (not shown) with the observations having higher temporal variability.

**Fig. 3:**
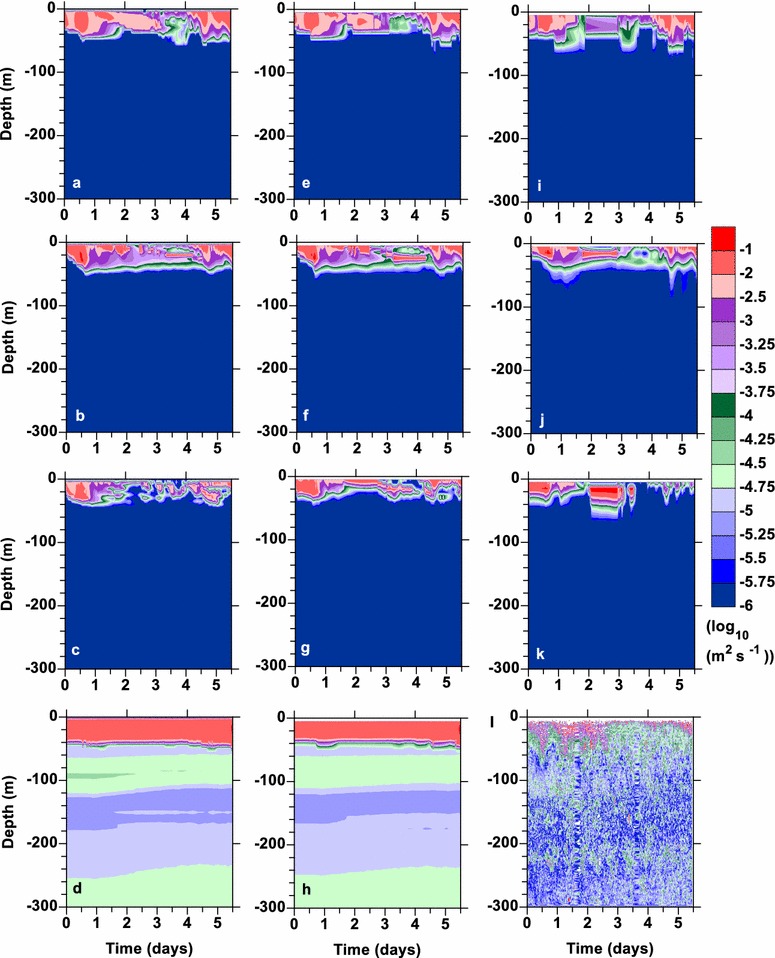
Log_10_ of the temperature diffusivities with depth during the FLX91 simulations (**a**–**k**) and the observations l. The simulations were done with (**a**–**d**) 100 levels, (**e**–**h**) 50 levels, and (**i**–**k**) 25 levels. The results from *Nakanishi*–*Niino* are given in (**a**, **e**, **i**), those from *Mellor*–*Yamada* 2.5 in (**b**, **f**, **j**), GLS in (**c**, **g**, **k**) and *Large*–*McWilliams*–*Doney* in (**d**, **h**). No results are shown for *Large*–*McWilliams*–*Doney* for 25 levels as they were essentially the same as those for 50 levels

Model estimates of temperature diffusivities varied widely between LMD and the other vertical mixing parameterizations (Fig. 3). LMD had much higher temperature diffusivities than the observations (Fig. 3l) at all vertical resolutions (Fig. 3d, h, 25 levels not shown). Temperature diffusivities generated by the simulations for the other mixing parameterizations (Fig. 3a–c, e–g, i–k) were higher near the surface and lower below 40 m than the observations (Fig. 3l) at all vertical resolutions. The observed temperature diffusivities are much more variable with time; however, the magnitudes are roughly equivalent, except below 40 m where the models are less diffusive. There are two reasons the models are less diffusive below 40 m. First, the stratification, as characterized by *N*, is too low for all resolutions (not shown). Second, the velocity shears are too low (not shown). The latter is due to the high vertical resolution of the observations compared to the model resolution, centimeters compared to meters. The shear stress at the surface from the wind matched between the observations and the model simulations for all vertical mixing parameterizations; however, this shear stress did not propagate vertically in the water column below 40 m in the model simulations. To see if the low diffusivity below 40 m affected the results, the background diffusivity was increased to 10^−5^ m^2^ s^−1^ for simulations with the NN and MY schemes with 100 levels. These simulations did not show appreciable differences in the smld or potential temperatures (not shown).

**Fig. 4:**
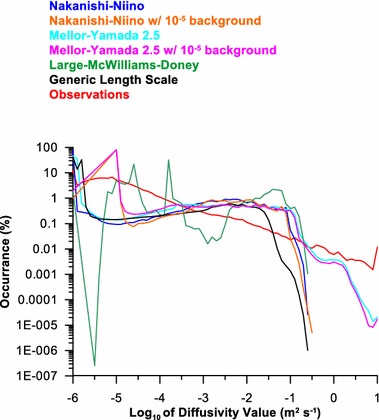
The distribution of the temperature diffusivities for the observations (red), *Nakanishi*–*Niino* (blue), *Mellor*–*Yamada* 2.5 (cyan), *Large*–*McWilliams*–*Doney* (green) and GLS (black) mixing schemes on log scales

Investigation of the distribution of the temperature diffusivities between the different vertical mixing parameterizations had a typical 2 peak distribution with 1 large peak near the molecular diffusivity 10^−6^ m^2^ s^−1^ and another between 10^−2^ and 10^−1^ m^2^ s^−1^ (Fig. 3). In the log distribution, the observations had a temperature diffusivity distribution, which generally decreased with larger magnitudes (red line in Fig. 4). The temperature diffusivity distributions for the simulations behaved differently. LMD had several peaks and a gap at low diffusivities (green line in Fig. 4). NN, MY, and GLS all had peaks between 10^−2^ and 10^−1^ m^2^ s^−1^ and NN and GLS roll off around 0.3 (10^−0.5^). MY responds most similarly to the observations; however, all mixing parameterizations are missing many values between 10^−6^ and 10^−4^ m^2^ s^−1^.

## Discussion

Although the model reproduced the surface mixed layer depth and potential temperatures reasonably well for the temperate, Eastern Pacific example in deep water, it did not reproduce the observed temperature diffusivities for the shallow, weaker stratification cases. The model estimates of temperature diffusivities were quite a bit higher than the observed values and lacked the high frequency variability of the observations. Increasing the vertical resolution did not affect the performance above the thermocline. In fact, the width of the thermocline was replicated more accurately with fewer vertical levels.

**Fig. 5:**
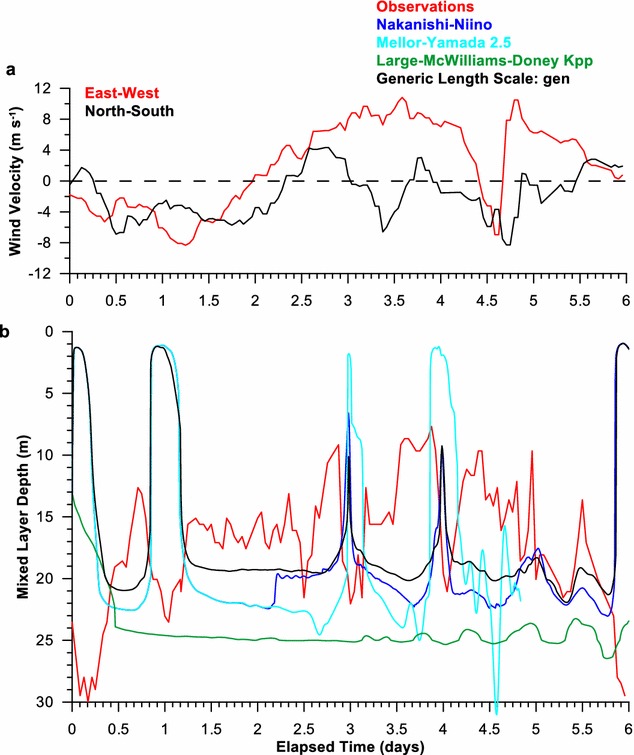
**a** The East–West (red) and North–South wind velocities during cruise 1 of the Baltic Sea simulations. **b** The Surface Mixed Layer Depth, smld, observations (red) and the model results for the *Nakanishi*–*Niino* (blue), Mellor–Yamada 2.5 (cyan), *Large*–*McWilliams*–*Doney* (green), and GLS (black) vertical mixing parameterizations

**Fig. 6:**
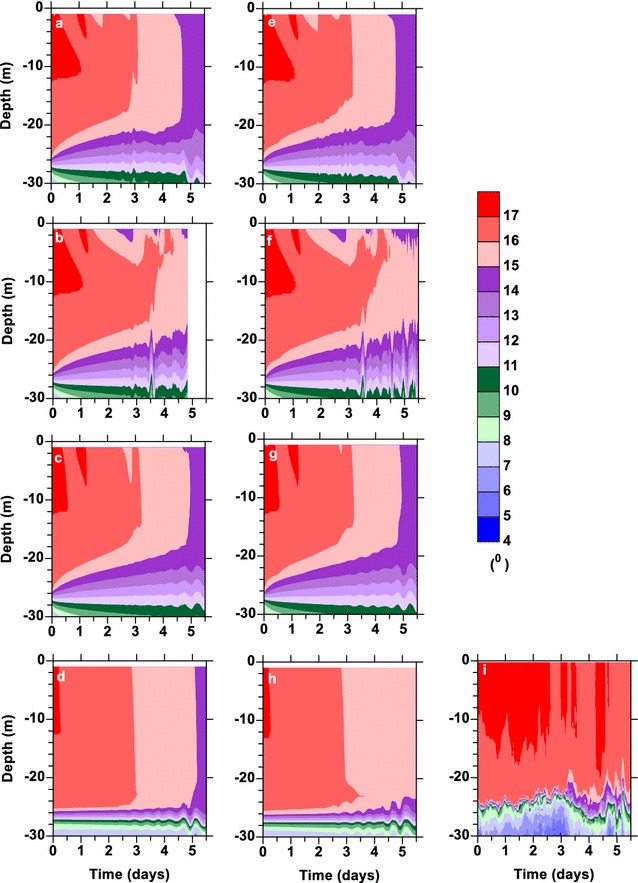
Potential temperature with depth during the simulations for the first Baltic Sea cruise (**a**–**i**) the observations. The simulations were done with (**a**–**d**) 50 levels and (**e**–**h**) 25 levels. The results from *Nakanishi*–*Niino* are given in (**a**, **e**), those from *Mellor*–*Yamada* 2.5 in (**b**, **f**), those from GLS in (**c**, **g**), and *Large*–*McWilliams*–*Doney* in (**d**, **h**)

In the shallow Baltic Sea, the surface mixed layer depths from the model were typically only a few meters deeper than that observed (Fig. 5b). However, the potential temperatures in the upper ocean become more mixed than the observations after a few days (Fig. 6). Winds for the Baltic Sea simulation were of similar strength as those during FLX91 (Fig. 5a), but the water depth was much shallower, ~ 50 m compared to ~ 4000 m. Here, all of the vertical mixing parameterizations overmixed the surface waters (Fig. 6), particularly in strong winds (Fig. 5a). This could be due to the shallow water, the weak stratification, numerical instabilities within the algorithm at high vertical resolution, or a combination of these factors. In the shallow waters, it is possible that the wind-generated mixed layer reaches the bottom or the mixed layer generated by bottom friction and the two become melded together. So the performance found for the FLX91 case did not hold for the Baltic Sea cases. In fact, MY had stability issues and failed mid-simulation for two of the three Baltic Sea cases (cyan line in Fig. 5b).

Overall, NN was the best performing, stable vertical mixing parameterization. MY performed similar to NN and better than GLS when the simulations were stable and completed. LMD mixed too much, which was also found for weak stratification by Durski et al. (2004). They also found that MY mixed more than LMD in strong stratification and less in weak stratification. Neither of these cases had really strong stratification. The surface mixed layer depth was reproduced by NN and MY and potential temperature fields for the FLX91 case, but not the three Baltic Sea cases, which experienced strong overmixing. None of the vertical mixing parameterizations reproduced the observed high frequency fluctuations of the temperature diffusivities. For their wind-driven mixing case, Durski et al. (2004) noted that neither MY nor LMD may be appropriate, indicating there was no clear best performer.

There is obviously room for improvement in the vertical mixing schemes. Their performance may be dependent on the wind and stratification conditions and we could be finding their limitations. The Baltic Sea may not be a good test case, due to its unique environmental conditions: low salinities, weaker stratification, shallow water depth, and strong winds. Obviously more observational data sets are needed to evaluate the performance in different wind and stratification conditions.

## Conclusions

The performances of four vertical mixing parameterizations, NN, MY, GLS, and LMD, were investigated at four observational sites. The model replicated potential temperatures and surface mixed layer depths reasonably well at the deep water site, but overmixed potential temperatures after a few days at the shallower site with weaker stratification. MY and NN performed the best, but MY occasionally failed at the shallow site, particularly in high winds. GLS had nearly the same performance as MY and NN, although underestimating the surface mixed layer depth by a few meters at the deep, temperate site. LMD performed the worst, generated the highest temperature diffusivities, and overmixed the most. The performance of the vertical mixing parameterizations appears to depend on stratification, wind strength, and possibly water depth. Further observational data sets are needed for comparison and more work needs to be done to determine the limitations of the parameterizations. There is definitely room for improvement with the vertical mixing parameterizations, particularly in their slow response, and with parameterizing the shear going into their estimates.

## Appendix A. Description of vertical mixing parameterizations

### A.1 *Mellor*–*Yamada* 2.5 level turbulence closure scheme (MY)

The *Mellor*–*Yamada* 2.5 level turbulence closure scheme (MY) was designed for boundary layer flows following the logarithmic law of the wall. It is based on equations for the turbulent kinetic energy and a length scale, both of which are time stepped through the simulation (Mellor and Yamada 1982). The parameterization was developed based on observations of laboratory turbulence. It is known to fail in the presence of stratification (Simpson et al. 1996) and was not designed for internal wave mixing (Large and Gent 1999).

### A.2 *Nakanishi*–*Niino* scheme (NN)

The *Nakanishi*–*Niino* scheme (NN) is adapted from the Mellor–Yamada 2.5 level turbulence closure scheme, in an effort to improve how the upper ocean, including the surface mixed layer is handled. The goal was take into account buoyancy effects on the pressure covariance terms affecting the coefficients used for turbulence closure. Like MY, NN is based on the turbulent kinetic energy and a length scale, which are time stepped through the simulation. The differences from MY primarily occur in determination of the length scale and the stability functions. In NN, the stratification plays a role in the calculation of the turbulent length scale. This results in the turbulent length scale decreasing with increasing density stratification (Furuichi and Hibiya 2015). This modification of the turbulent length scale based on buoyancy does not occur in MY (Furuichi et al. 2012). Another difference between MY and NN occurs with the stability functions, where NN includes buoyancy effects in the pressure covariance terms used to determine the constants. A fuller description of the differences, including the mathematics, is given in Furuichi et al. (2012) and Furuichi and Hibiya (2015). To evaluate the performance of NN vs MY in the ocean, Furuichi and Hibiya (2015) compared microstructure observations to model results, using a different model than ROMS. They found NN better matched the observations.

### A.3 *Large*–*McWilliams*–*Doney* Kpp scheme (LMD)

*LMD* separates mixing physics into three primary physical processes: local *R*_*i*_ instabilities due to resolvable vertical shear, internal wave, and double diffusion. The total vertical mixing coefficient for momentum, *K*_*ν*_, is a sum of coefficients resolvable vertical shear and internal waves *K*_*ν*_ = *K*_*νs*_ + *K*_*νi*_, while the total vertical mixing coefficient for tracers, *K*_*T*_, is a sum of coefficients for each of the three processes *K*_*T*_ = *K*_*Ts*_ + *K*_*Ti*_ + *K*_*Td*_. For *R*_*i*_ < 0.8, the first of these processes dominates and induces a *R*_*i*_ dependence $$\mathop K\nolimits_{\nu s} = \mathop {10}\nolimits^{ - 3} \mathop {\left( {1 - {\raise0.7ex\hbox{${\mathop R\nolimits_{i} }$} \!\mathord{\left/ {\vphantom {{\mathop R\nolimits_{i} } {0.7}}}\right.\kern-0pt} \!\lower0.7ex\hbox{${0.7}$}}} \right)}\nolimits^{3}$$ m^2^ s^−1^. For *R*_*i*_ > 0.8, the internal wave term dominates and *K*_*ν*_ is a dependent on *N* according to *K*_*ν*_ = 10^−6^/10^−6^ N^2^ N^2^ m^2^ s^−1^ with a maximum cutoff value of ~ 1.0 × 10^−4^ m^2^ s^−1^. In addition, a *K* profile is applied both at the surface and the bottom to represent surface and benthic boundary layers, respectfully. Care was taken in the development of this scheme to avoid scale dependencies (*W. Large*, personal communication, 2004).

### A.4 generic length scale scheme (GLS)

Umlauf and Burchard (2003) evaluated different mixing parameterizations and developed a set of generic equations, which could be used to describe the combined contribution of various mixing processes:

1 $${{\partial \psi } \mathord{\left/ {\vphantom {{\partial \psi } {\partial t}}} \right. \kern-0pt} {\partial t}} + \mathop u\nolimits_{i} {{\partial \psi } \mathord{\left/ {\vphantom {{\partial \psi } {\partial \mathop x\nolimits_{i} }}} \right. \kern-0pt} {\partial \mathop x\nolimits_{i} }} = \mathop D\nolimits_{\psi } + \frac{\psi }{\kappa }\left( {\mathop c\nolimits_{\psi 1} P - \mathop c\nolimits_{\psi 2} \varepsilon + \mathop c\nolimits_{\psi 3} G} \right)$$

2 $$\psi = \mathop {\left( {\mathop c\nolimits_{\mu }^{o} } \right)}\nolimits^{p} \mathop \kappa \nolimits^{m} \mathop \varepsilon \nolimits^{n}$$

where *D* represents the turbulent and viscous transport, *P* the kinetic energy production by shear, *G* the kinetic energy production by buoyancy, *ε* the dissipation, *κ* the turbulent kinetic energy, *c*’s model constants, and *m*, *n*, and *p* exponents. Like *MY*, the turbulent kinetic energy and length scale are time stepped. In fact *MY* is a special case of *GLS* with *m* = 1*, n* = 1, and *p* = 0. Special cases of *GLS* represent two other developed parameterizations, *κ* − *ε* (*GLS*: *κ* − *ε)* and *κ* − *ω* (*GLS*: *κ* − *ω)* (*GLS*: *κ* − ε: *m* = 1.5*, n* = − 1, and *p* = 3; *GLS*: *κ* − ω: *m* = 0.5*, n* = − 1, and *p* = − 1). In the *κ* − *ε* parameterization, the dissipation, *ε*, is used for the length scale. This parameterization was developed by Jones and Launder (1972) and recently modified by Burchard and Bolding (2001). In the *κ* − *ω* parameterization, the rate of dissipation of energy per unit volume and time, *ω*, is used as the length scale, with $\omega = \frac{\varepsilon }{{\mathop {\left( {\mathop c\nolimits_{\mu }^{0} } \right)}\nolimits^{4} k}}$ where $c_{\mu }^{0}$ again is a constant. In order to use this scheme in stratified flows, buoyancy was included by Umlauf et al. (2003). An additional scheme (*GLS*: *gen*) with *m* = 1*, n* = − 0.67, and *p* = 2 was developed by Umlauf and Burchard (Warner et al. 2005). The standard values for the generic option as given in the in-file description were used. Readers interested in understanding more about the different length scale assumptions and details of the parameterizations are encouraged to consult the ROMS website (https://www.myroms.org/) and papers by Burchard, Umlauf and others (Burchard et al. 1998; Burchard and Bolding 2001; Umlauf and Burchard 2003; Umlauf et al. 2003). *GLS* has been implemented in ROMS. For this study, the *GLS* parameters used were those for generic scheme. Warner et al. (2005) compared the various *GLS* schemes for various applications including steady barotropic flow, wind-induced surface mixed layer deepening in a stratified fluid, oscillatory stratified pressure-gradient driven flow, and an estuarine situation. The *GLS*: *κ* − *κl* performed poorly in the estuarine flow and the other three, *GLS*: *κ* − *ε*, *GLS*: *κ* − *ω*, and *GLS*: *gen*, performed similarly with slight differences (Warner et al. 2005). They were unable to ascertain which of these three *GLS*: *κ* − *ε*, *GLS*: *κ* − *ω*, and *GLS*: *gen*, was most realistic (Warner et al. 2005).
